# STI in times of PrEP: high prevalence of chlamydia, gonorrhea, and mycoplasma at different anatomic sites in men who have sex with men in Germany

**DOI:** 10.1186/s12879-020-4831-4

**Published:** 2020-02-07

**Authors:** Klaus Jansen, Gyde Steffen, Anja Potthoff, Ann-Kathrin Schuppe, Daniel Beer, Heiko Jessen, Stefan Scholten, Petra Spornraft-Ragaller, Viviane Bremer, Carsten Tiemann, MSM Screening Study group, Heribert Knechten, Petra Panstruga, Daniel Beer, Christiane Cordes, Christian Lieb, Elena Rodriguez, Heribert Hillenbrand, Heiko Jessen, Ivanka Krznaric, Anja Potthoff, Martin Hower, Petra Spornraft-Ragaller, Stefan Scholten, Stephan Schneeweiß, Anja Meurer, Ramona Pauli, Andrea Tomesch, Andreas Trein

**Affiliations:** 1 Robert Koch Institute, Unit for HIV/AIDS, STI and Blood-borne Infections, Department for Infectious Disease Epidemiology, Seestrasse 10, 13353 Berlin, Germany; 2 Postgraduate Training for Applied Epidemiology (PAE), Robert Koch Institute, Berlin, Germany; 3 European Programme for Intervention Epidemiology Training (EPIET), European Centre for Disease Prevention and Control (ECDC), Stockholm, Sweden; 4 Walk in Ruhr (WIR), Centre for Sexual Health and Medicine, Clinic of the Ruhr University, Bochum, Germany; 5 Labor Krone/Labcon-OWL, Bad Salzuflen, Germany; 6 Praxis/Labor Dr. med. Heribert Knechten, Aachen, Germany; 7 Praxis Jessen2, Berlin, Germany; 8 Praxis Hohenstaufenring, Köln, Germany; 9 Department of Dermatology, University Hospital Carl Gustav Carus, Technical University of Dresden, Dresden, Germany

**Keywords:** MSM, Prevalence, PrEP, HIV, Chlamydia trachomatis, *Neisseria gonorrhoeae*, Mycoplasma genitalium

## Abstract

**Background:**

Men who have sex with men (MSM) are disproportionally affected by sexually transmitted infections (STI). STI are often extragenital and asymptomatic. Both can delay diagnosis and treatment. Approval of HIV pre-exposure prophylaxis (PrEP) might have influenced sexual behaviour and STI-prevalence of HIV- MSM. We estimated STI-prevalence and risk factors amongst HIV- and HIV+ MSM in Germany to plan effective interventions.

**Methods:**

We conducted a nationwide, cross-sectional study between February and July 2018. Thirteen MSM-friendly STI-practices screened MSM for *Chlamydia trachomatis (CT)*, *Mycoplasma genitalium* (MG), *Neisseria gonorrhea (NG)*, and *Trichomonas vaginalis* (TV) using self-collected rectal and pharyngeal swabs, and urine samples. APTIMA™ STI-assays (Hologic™ Inc., San Diego, USA) were used for diagnostics, and samples were not pooled. We collected information on socio-demographics, HIV-status, clinical symptoms, sexual behaviour within the last 6 months, and PrEP use. We combined HIV status and PrEP use for defining risk groups, and used directed acyclic graphs and multivariable logistic regression to identify risk factors for STI.

**Results:**

Two thousand three hundred three MSM were included: 50.5% HIV+, median age 39 [18–79] years. Median number of male sex partners within the last 6 months was five. Sex without condom was reported by 73.6%, use of party drugs by 44.6%. 80.3% had a STI history, 32.2% of STI+ MSM reported STI-related symptoms. 27.6% of HIV- MSM used PrEP.

Overall STI-prevalence was 30.1, 25.0% in HIV−/PrEP- MSM (CT:7.2%; MG:14.2%; NG:7.4%; TV:0%), 40.3% in HIV−/PrEP+ MSM (CT:13.8%; MG:19.4%; NG:14.8%; TV:0.4%), and 30.8% in HIV+ MSM (CT:10.1%; MG:18.4%; NG:8.6%; TV:0.1%).

Being HIV+ (OR 1.7, 95%-CI 1.3–2.2), using PrEP (OR 2.0, 95%-CI 1.5–2.7), having > 5 sex partners (OR:1.65; 95%-CI:1.32–2.01.9), having condomless sex (OR:2.11.9; 95%-CI:1.65–2.86), and using party drugs (OR:1.65; 95%-CI:1.32–2.0) were independent risk factors for being tested positive for at least one STI.

**Conclusions:**

We found a high STI-prevalence in MSM in Germany, especially in PrEP users, frequently being asymptomatic. As a relevant proportion of PrEP users will not use a condom, counselling and comprehensive STI screening is essential and should be low threshold and preferably free of cost. Counselling of PrEP users should also address use of party drugs.

## Background

Men who have sex with men (MSM) are disproportionally affected by sexually transmitted infections (STI), such as *Chlamydia trachomatis* (CT), *Neisseria gonorrhoeae* (NG), or syphilis [1–7]. STI are often asymptomatic, and therefore remaining frequently undetected and untreated [8]. This may lead to severe sequelae, and serve as ongoing transmission reservoir. Extragenital STI in MSM are frequent [8–12] and can contribute substantially to the further spread if not diagnosed and treated. In previous studies, HIV-positive (HIV+) MSM often showed higher prevalences of STI than HIV-negative (HIV-) MSM [13, 14]. As reasons for higher STI prevalence in MSM in general, higher number of sexual partners as well as higher frequencies of sexual practices with higher risk for acquiring STI are discussed [3, 6, 7, 14]. In Germany, medical guidelines recommend risk adapted STI testing for MSM [15], but costs are not covered by German health insurance if patients do not show STI-related symptoms or if there is no clear report of a substantial risk of infection. In these cases, patients have to bear costs for STI testing privately or physicians risk claim for damages by balancing accounts with insurance companies for testing asymptomatic patients. Therefore the scope of asymptomatic, undetected and potentially transmissible STI in MSM in Germany remains still unknown and may be high.

In 2016 pre-exposure prophylaxis (PrEP) against HIV infection was approved in Germany. For PrEP, tenofovir disoproxil fumarate and emtricitabine is taken by patients preferably as daily oral medication, showing high effectivity against HIV infection [16–21]. MSM with increased sexual risk behavior and/or recent STI are eligible for PrEP according to WHO guidelines as well as guidelines of the German-Austrian medical AIDS society [22, 23]. The latter recommend syphilis testing for PrEP users every 3 months, and testing for CT and NG every three to 6 months. PrEP users in Germany had to bear the expenses for PrEP and all corresponding tests (HIV, STI, creatinine) privately until recently. Since September 2016, several generic medicaments for PrEP were available and reduced costs distinctly (ca. 50€/month), leading to a broader implementation of PrEP in Germany. Since September 1st 2019, German compulsory health insurance covers the costs of PrEP and related testing of necessary clinical parameters and STI (ca. 90% of the population). The frequency of testing as well as its extent will be individually defined by the treating physician according to risk behavior and/or symptoms and will be covered by the health insurance for the evaluation phase of PrEP in Germany [24].

Despite the license of PrEP includes the recommendation of regular condom use for PrEP users, it is probable that one of the main reasons for taking PrEP is that persons can effectively reduce their risk for acquiring a HIV infection without using condoms. Since PrEP was introduced it is under debate to what extent a concomitant reduction of condom use and a potential increase of more risky sexual behavior will lead to an increase of other STI [25–30]. In contrast, recommended regular and small meshed STI testing is discussed as an argument against an increase of STI due to PrEP, as this could lead to more efficient diagnosis and treatment of newly acquired STI as well as of so far undiagnosed reservoirs in populations with high risk such as sexually highly active MSM [31, 32]. Subsequently, a reduction of STI prevalence could result in the medium and long term.

As the national approval of PrEP in 2016 may have an influence on sexual behavior and STI prevalence in MSM in general, the need of systematic data on STI prevalence in MSM is urgent to estimate their STI risk, to provide reliable data to define appropriate testing algorithms for MSM using PrEP or not, and to plan effective preventional measures for PrEP using MSM and all other MSM at risk for STI.

With the “MSM Screening Study” we aimed estimating the current prevalence of CT, NG, Mycoplasma genitalium (MG) and Trichomonas vaginalis (TV) as well as relevant risk factors among the general MSM population (HIV+ and HIV-) in Germany and to compare STI prevalences systematically by HIV status, PrEP use and localization.

## Methods

### Study type

Between February and August 2018, we conducted a nationwide cross-sectional multicentre study to estimate the prevalence of CT, MG, NG and TV in MSM in nine large cities across Germany (Aachen, Berlin, Bochum, Cologne, Dortmund, Dresden, Munich, Nuremberg, and Stuttgart).

### Setting, study population

**Fig. 1:**
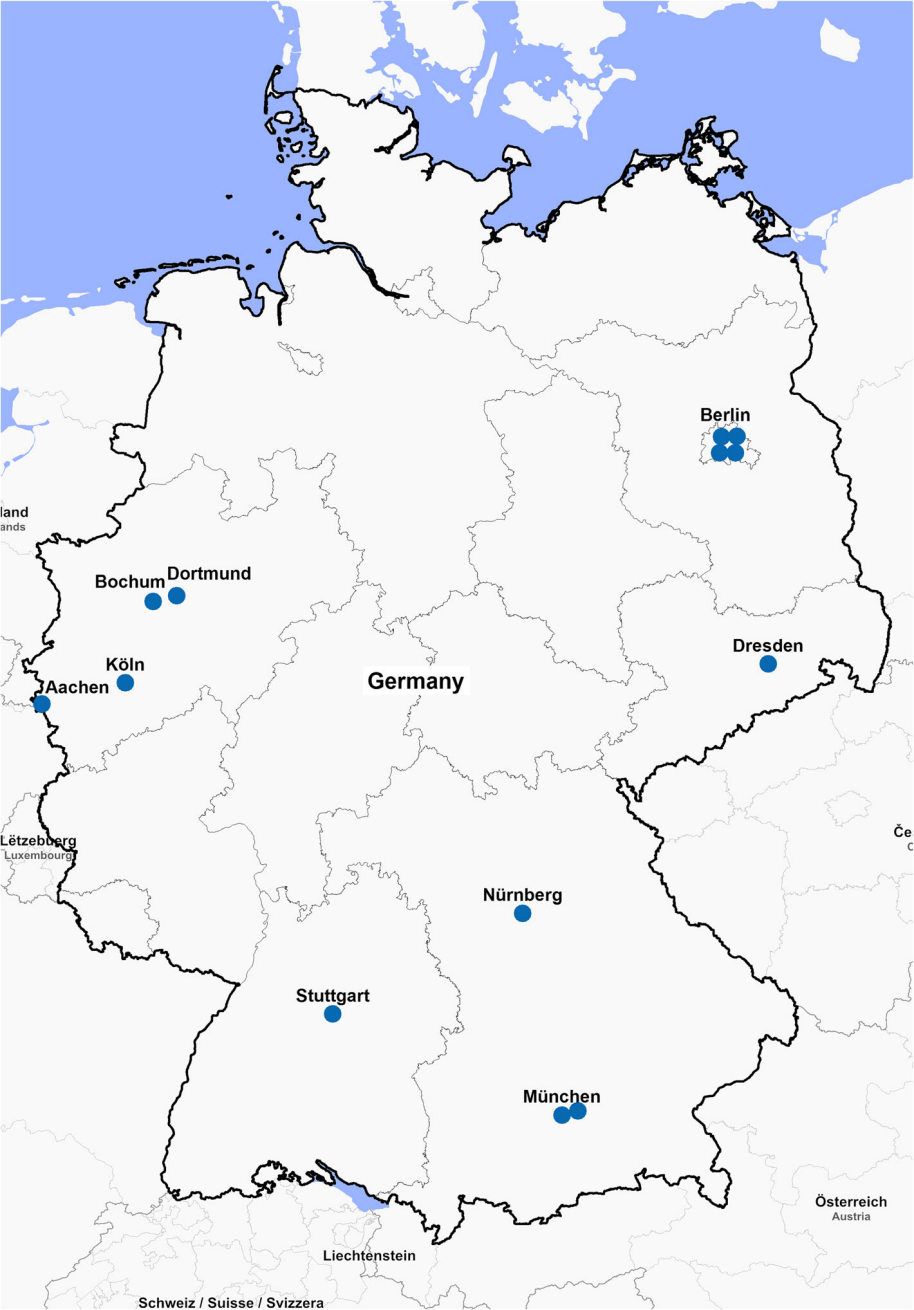
Geographical distribution of study sites of the MSM Screening Study (map authors' own)

For the study we recruited a convenience sample of MSM-friendly practices with infectiological focus that were frequently visited by HIV-positive and HIV-negative MSM, due to their profile also serving as general practitioners for MSM. Of 30 requested sites, 13 participated in the study (Fig. 1).

Within the testing period all MSM attending the testing site were invited to participate in the study, independent from the reason of their visit or any symptoms. The participants did not receive any incentive for taking part in the study.

#### Inclusion criteria

Inclusion criteria were: age >=18 years, no former participation in the MSM Screening Study, known HIV-status (HIV test result within the last 12 months), no antibiotic STI therapy within the last 4 weeks and informed consent to take a pharyngeal and rectal swab and to provide a urine sample to be tested for CT, NG, MG and TV.

### Data and samples collected

#### Study questionnaire

The study participants filled in a standardised self-administered questionnaire that was designed specifically for the study (Additional file 1). It consisted of 20 questions gathering information on sociodemographics, sexual behaviour and use of drugs (alcohol, Cannabis, Heroine, Poppers, Cocaine/Speed, Ecstasy, Viagra/Cialis, Speed, GLB/GHB, Crystal Meth, Bath salts/ Spice) in the last 6 months, STI-related symptoms in the last 4 weeks, STI history, HIV status (plus where applicable information on HIV therapy and outcome) and current use of PrEP. The HIV-status was also obtained from the testing site.

#### Sample collection and diagnostic tests

Biological samples were obtained using rectal and pharyngeal swabs, and urine samples. Samples were self-collected (with Aptima™ Multitest Swab Specimen Collection Kit and Aptima™ Urine Specimen Collection Kit), after instruction by the medical staff of the testing site and using a photograph -based demonstration material especially developed for the MSM Screening Study.

The samples were not pooled and tested by transcription-mediated amplification with the Hologic™ (Hologic Inc., San Diego, USA) APTIMA Combo 2™ Assay for CT and NG; the APTIMA™ Mycoplasma genitalium Assay for MG and the APTIMA™ Trichomonas vaginalis Assay for TV, using the Hologic™ Panther System.

### Statistical analysis

With an estimated STI prevalence of 6% among HIV-negative and 12% among HIV-positive MSM, a power of 80% and a precision of 2 to 3%, a study population size of 1200 HIV- and 980 HIV+ participants was needed for sufficient prevalence estimations related to HIV-status. To ensure sufficient statistical power prevalence estimations for both, HIV- and HIV+ MSM, HIV+ MSM were oversampled compared to their proportion of the overall MSM population in Germany. With an estimated response rate of 70%, 1700 HIV-negative and 1400 HIV-positive MSM had to be invited for participation in the study.

We described the study population calculating frequencies and proportions for dichotomous and categorical variables and the median for continuous variables (age). We calculated the overall prevalence for CT, NG, MG and TV including 95% confidence intervals (95%-CI), and tested bivariable correlations between sociodemographic/behavioural factors and STI prevalence using chi-squared test and Wilcoxon-Mann-Whitney test as appropriate.

We stratified for HIV-status for prevalence calculations. Additionally, we combined HIV and PrEP use to define meaningful risk profiles. The three different risk groups were HIV positive MSM (HIV+), HIV-negative non-PrEP users (HIV−/PreP-) and HIV-negative PrEP users (HIV−/PrEP+).

As basis for the multivariable analyses, we used directed acyclic graphs (DAGs) [33] to explore the potential causal relationships between the risk groups, sexual behaviour and being tested positive for at least one STI considering several co-variates. Moreover we identified minimally sufficient adjustment sets to minimize confounding. As sexual behaviour was shown to be on the causal path between PrEP use/ HIV status and STI status, we developed two separate multivariable regression models. First, we investigated statistical associations between the three risk groups and the outcome “tested positive for at least one STI”, additionally sub-analysing the influence of HIV status (excluding PrEP users) and PrEP use (excluding HIV-positive MSM) on tested positive for at least one STI separately. In a second step, we estimated associations between sexual behaviour and the same outcome. Differences in sexual behaviour between the risk groups were analysed descriptively.

For multivariable analyses we used manually stepwise forward selected logistic regression calculating odds ratios (OR). We tested all eligible factors bivariable associated with the outcome at *p* < 0.2. The overall significance level was set at *p* < 0.05.

All analyses were performed using STATA V.14 software package (StataCorp LP, College Station, Texas, USA).

### Data protection

A unique identifier (barcode) was used to allocate samples and questionnaires to the participants. The testing sites received the test results and could link them via barcode to their patients. At the laboratory, the barcode was removed from the datasets after linking of the laboratory data with the data of the questionnaire. The Robert Koch Institute received a completely anonymised dataset for analysis.

Test results were communicated from the laboratory to the testing sites within 24 h. All participants tested positive for any of the measured STI were informed and consulted by their attending physician and could receive treatment by their testing site based on the national STI treatment guideline.

## Results

### Demographic characteristics and sexual behaviour of the study population

Between 20/2/2018 and 2/7/2018, 2321 MSM participated in the study, between 32 and 312 MSM by each site. Complete test results for all four pathogens and information on HIV-status were available for 2303 of them, constituting the final study population. 50.5% (1164/2303) of all participants were HIV+. 91.4% of them were diagnosed with HIV longer than 12 months before study entry, 98.4% were on antiviral treatment and 84.2% reported a viral load below the detection limit. Of the HIV- participants, 27.6% (283/1024) reported current PrEP use (HIV−/PrEP+), 72.4% (741/1024) did currently not taking PrEP (HIV−/PrEP-).

34.2% of all participating MSM were between 30 and 40 years old (Table 1), HIV+ MSM were older than HIV−/PrEP- and HIV−/PrEP+ (median 44 [20–79] vs. 34 [18–73] and 35 [20–66]). Participants not being born in Germany (25.9%) came from various countries all over the world, mostly from Brazil (7.4% of foreign born MSM), United States of America (6.4%), Italy (6.0%), and Poland (5.5%). HIV−/PrEP+ were more often born abroad (37.2%) than HIV−/PrEP- (30.9%) and HIV+ (19.2%). HIV−/PrEP+ and HIV−/PrEP- had more often a university-entrance diploma (80.2 and 79.5%) than HIV+ (54.4%).

**Table 1:** Sociodemographic, behavioral and clinical characteristics of the study population, by HIV/PrEP-status

N^b^	ALL	HIV+	HIV−/PrEP-	HIV−/PrEP+
	2303^a^	1164	745	283
Demographics^c^				
Age (median [range]; *n* = 2287)	39 [18–79]	44 [20–79]	34 [18–73]	35 [20–66]
Born in Germany (%; *n* = 2168)	74.5	80.8	69.1	62.8
University-entrance diploma (%; *n* = 2157)	66.2	54.4	79.5	80.2
Sexual behavior in the last 6 months				
Number of sex partners (median [range]; *n* = 1935)	5 [0–820]	4 [0–820]	5 [0–120]	11 [1–240]
Sex without condom (%, *n* = 2148)	73.6	73.1	67.3	91.8
Condomless anal intercourse (insertive; %; *n* = 2076)	55.7	56.8	51.5	78.4
Condomless anal intercourse (receptive; %; *n* = 2077)	59.2	63.7	46.6	73.8
Condomless oral intercourse (insertive; %; n = 2077)	87.2	83.9	89.6	94.0
Condomless oral intercourse (receptive; %; *n* = 2078)	81.2	76.1	85.9	88.7
Rimming (active; %; *n* = 2075)	54.9	49.3	56.0	73.1
Rimming (passive; %; n = 2076)	58.1	52.4	62.1	77.0
Fisting (active; %; *n* = 2072)	15.5	16.0	11.8	23.0
Fisting (passive; %; n = 2072)	9.6	11.7	5.9	11.7
Use of drugs (%; *n* = 2123)	67.7	64.3	66.6	84.2
Use of party drugs (%; *n* = 2123)	44.6	42.9	39.6	64.4
Paid for sex (%; *n* = 1814)	3.5	3.7	3.4	2.7
Being paid for sex (%; *n* = 1791)	2.8	2.9	2.9	2.6
STI history				
STI in medical history (%, *n* = 1908)	80.3	96.6	59.4	81.1
Chlamydia trachomatis (%, n = 2148)	39.8	42.6	30.9	52.1
Hepatitis B (%, *n* = 2144)	11.0	16.7	4.0	5.7
Hepatitis C (%, *n* = 2145)	8.6	15.0	1.2	1.8
Mycoplasma genitalium (%, *n* = 2139)	6.9	5.8	5.8	14.2
*Neisseria gonorrhoeae* (%, *n* = 2188)	46.3	48.6	37.9	58.9
Treponema pallidum (%, *n* = 2155)	40.9	55.4	20.8	33.7
Report of STI-related clinical symptoms in the last four weeks (%; *n*=;642)	32.2	29.1	37.7	33.0

^a^The total number of study participants also includes HIV-negative MSM with no data given on their PrEP use

^b^The N represents the number of participants in each group

^c^The n represent the number of participants answering the question

Most participating MSM reported to be single (44.8%) or to live in an open relationship with an agreement for sex with others (32.6%). Most stated to have met their sex partners on the internet (77.6%), in bars (36.8%) or in saunas (28.4%). The proportion of singles and MSM in open relationships was higher in HIV−/PrEP+ (96.8%) than in HIV−/PrEP- and HIV+ MSM (82.9 and 76.7%, respectively). 44.9% of all participants reported more than five male sex partners during the last 6 months, the proportion was higher in HIV−/PrEP+ (79.8%) than in HIV−/PrEP- (46.1%) and HIV+ (36.4%). Condomless anal intercourse (CAI, insertive and/or receptive) was reported by 73.2%, and more frequently from participating MSM reporting more than five sex partners (84.6%) than from MSM reporting one to four sex partners (66.5%), and more frequently from HIV−/PrEP+ (91.8%) than from HIV−/PrEP- (67.3%) and HIV+ MSM (73.1%).

The most frequently used risk reduction strategies to avoid HIV-infection when not using condoms were to ask the partner for his HIV-status (40%), only to have sex with HIV+ partners if they have an undetectable viral load (26.2%), only to have sex without a condom in a monogamous relationship (20.3%) and to use PrEP (15.6%).

The mainly reported substances used in the context of sexual encounters within the last 6 months were alcohol (80.6%), Poppers (53.9%), Viagra/Cialis (33.9%), and Cannabis (31.9%). 43.5% of all participants reported to use so called party drugs (defined as Cocaine, Crystal Meth, Ecstasy, GBL/GHB, Mephedron/Spice, Poppers and Speed) in the context of sexual encounters within the last 6 months; the use of party drugs was higher in HIV−/PrEP+ (64.4%) than in HIV−/PrEP- (39.6%) and HIV+ MSM (42.9%). The detailed population characteristics are summarized in Table 1.

HIV- participants were significantly younger than HIV+ MSM (median 35 IQR [30–43] vs. 44 [35–52], *p* < 0.01) and less likely to be born in Germany (67.2% vs. 80.8%, *p* < 0.01), but they were more likely to have acquired university-entrance diploma (74.6% vs. 54.4%, *p* < 0.01). The median number of male sex partners was significantly higher in HIV- MSM than in HIV+ (6 [3–15] vs. 4 [1–10], *p* < 0.01). There was no difference in having sex without using condoms in the last 6 months (73.1% vs. 73.4%, *p* = 0.88). The proportion of reported insertive condomless anal intercourse (CAI) did not differ between HIV- and HIV+ MSM (58.4% vs. 56.8%, *p* = 0.460). HIV- participants reported more insertive condomless oral intercourse (COI) (90.0% vs. 83.9%, *p* < 0.05) and receptive COI (86.0% vs. 76.1%, *p* < 0.05), but less receptive CAI (52.8% vs. 63.7%, *p* < 0.05). The use of party drugs did not differ between both groups (44.2 vs. 42.9%, *p* = 0.56). The proportion of participants with a STI history was significantly lower in HIV- participants (64.0 vs. 96.6%, *p* < 0.01). The proportion of clinical symptoms among positive STI-tested participants did not differ between both (35.4 vs. 29.1%, *p* = 0.08).

Regarding sociodemographics HIV−/PrEP- and HIV−/PrEP+ did not significantly differ. The differences between HIV+ and HIV- participants are described above. HIV−/PrEP+ reported the highest number of male sex partners (median 11 IQR [6–25]) and the highest proportion of sex without using condoms (91.8%), including insertive and receptive CAI (78.4, 73.8%) and COI (94.0, 88.7%). Also they reported to more frequently use party drugs (64.4%). The report of symptoms in positive tested participants was highest in HIV−/PrEP- (37.7%), followed by HIV−/PrEP+ (33.0%) and HIV+ (29.1%). The proportion of a previous STI was highest in HIV+ MSM (96.6%), followed by HIV−/PrEP+ (81.1%) and lowest in HIV−/PrEP- (59.4%).

### Prevalence of CT, MG, NG, and TV

All together 30.1% (693) of all participants were tested positive for at least one of the tested STI (for specific prevalences, see Table 2), MG was the most prevalent pathogen (17.0%), TV was diagnosed only in 2 participants (Table 2).

**Table 2:** Prevalence of Chlamydia trachomatis, Neisseria gonorrhoeae, Mycoplasma genitalium and Trichomonas vaginalis, by pathogen and anatomical location

*N* = 2203	Any STI			CT			NG			MG			TV			Multiple pathogens		
	n	%	95%-CI	n	%	95%-CI	n	%	95%-CI	n	%	95%-CI	n	%	95%-CI	n	%	95%-CI
Any site	693	30.1	28.2–32.0	227	9.9	8.7–11.1	205	8.9	7.8–10.1	391	17	15.5–18.6	2	0.1	0.01–0.3	117	5.1	4.2–6.0
Pharynx	192	8.3	7.2–9.5	26	1.1	0.7–1.6	110	4.8	3.9–5.7	66	2.9	0.2–0.3	0	0	0	48	2.1	1.5–2.7
Rectum	503	21.8	20.2–23.6	178	7.7	6.7–8.9	133	5.8	4.8–6.8	265	11.5	10.2–12.9	2	0.1	0.01–0.3	102	4.4	3.6–5.3
Urine	192	8.3	7.2–9.5	45	2.0	1.4–2.6	32	1.4	0.9–1.9	124	5.4	4.5–6.4	0	0	0	48	2.1	1.5–2.7
Multiple sites	170	7.4	6.3–8.5	63	2.7	2.1–3.5	91	3.9	3.2–4.8	99	4.3	3.5–5.2	0	0	0	72	3.1	2.4–3.9

16.9% (117) of participants were tested positive for more than one pathogen, of those 35.9% [34] for CT/MG, 28.2% [33] for CT/NG, 23.9% [28] for NG/MG, and 12.0% [14] for CT/MG/NG.

50.6% (351) of all diagnosed STI were solely manifested rectal, 11.1% (77) pharyngeal, 13.7% (95) urethral, and 24.5% (170) of all infections were manifested in more than one anatomical location. While for CT and MG the prevalence was lowest for pharyngeal infections, for NG the prevalence for pharyngeal infections was higher than for urogenital infections (Table 2). Rectal MG exhibited the highest prevalence (11.5%) of all diagnosed STI.

### Clinical symptoms of STI-positive participants

About a third of all participants reported STI-related clinical symptoms in the last 4 weeks, this differed slightly between risk groups (Table 1). The proportion of STI-positive diagnosed participants that reported clinical symptoms was 32.1% overall, and highest in participants with urogenital only infections (37.0%), followed by rectal only (28.3%) and oral only infections (24.0%). The proportion of reported symptoms in participants with multiple site infections was 41.1%. Stratified by pathogen, 29.3% of all only CT-positive participants, 40.9% of all only NG-positive participants, and 29.3% of all only MG-positive participants reported clinical symptoms.

### Impact of HIV status and PrEP use on STI prevalence

#### HIV status

The overall STI prevalence did not significantly differ between HIV- and HIV+ participants (30.8% vs. 29.4%, *p* = 0.48), as did not the single prevalences of CT (10.1% vs. 9.6%, *p* = 0.65), NG (8.6% vs. 9.2%, *p* = 0.60), and MG (18.4% vs. 15.5%, *p* = 0.07). The overall STI prevalence did not significantly differ between HIV+ participants having a HIV viral load below the detection limit (29.2% vs. 33.3%, *p* = 0.46) or above.

#### Risk groups HIV+ MSM, HIV−/PrEP- MSM, HIV−/PrEP+ MSM

**Fig. 2:**
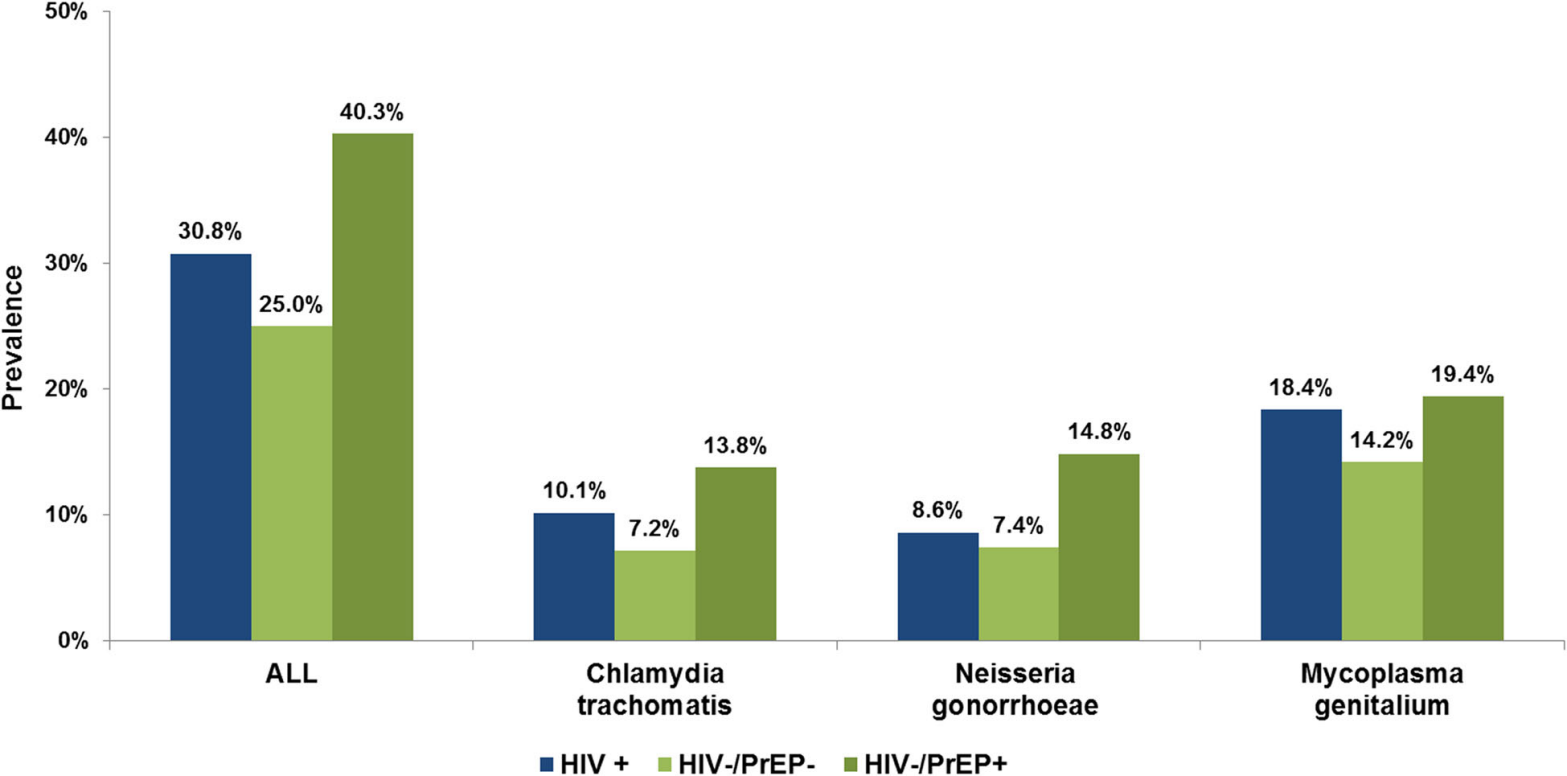
Prevalence of *Chlamydia trachomatis*, *Neisseria gonorrhoeae* and *Mycoplasma genitalium*, by HIV/PrEP-status (*n* = 2303)

**Fig. 3:**
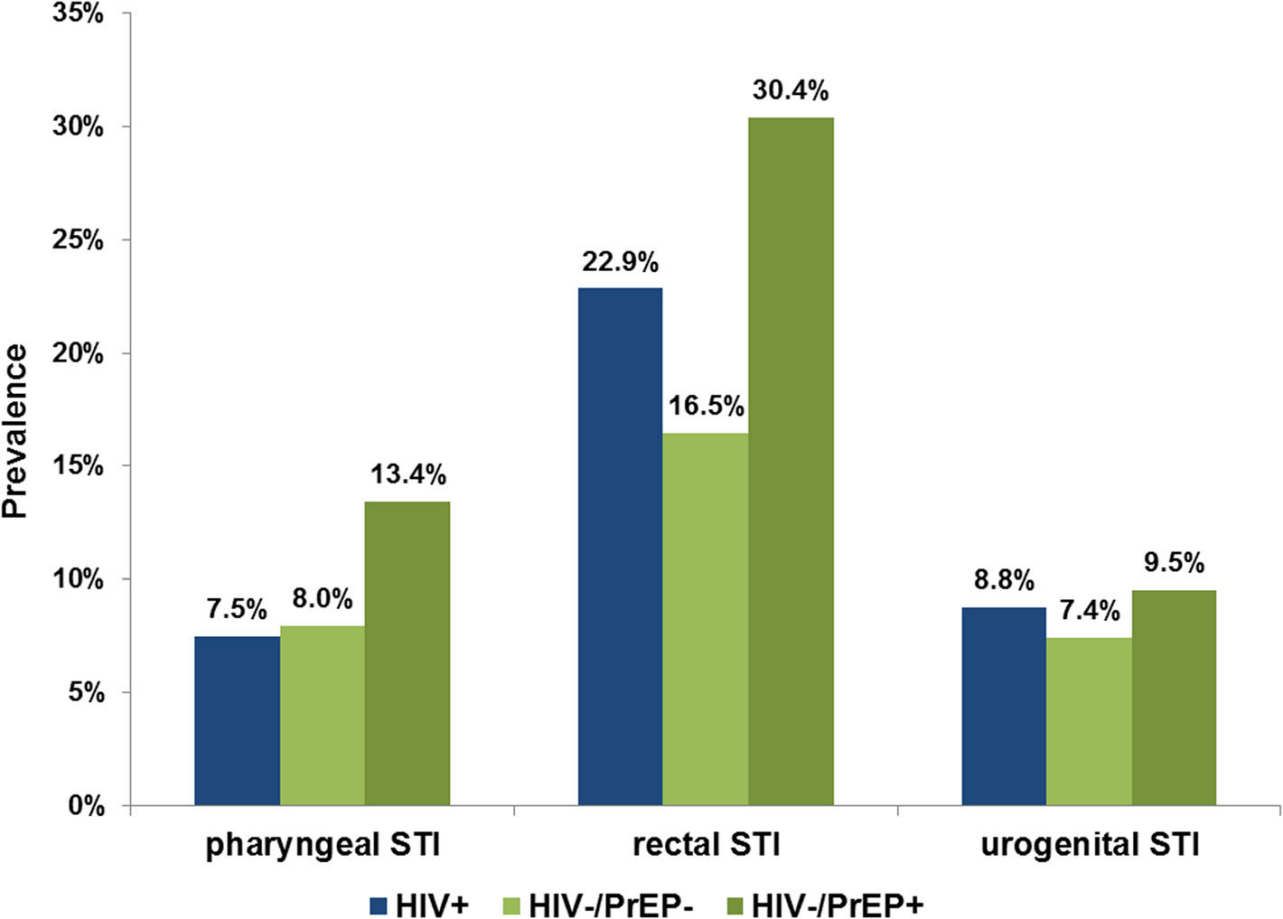
Prevalence of any STI, by anatomical location and HIV/PrEP-Status (*n* = 2303)

The overall STI prevalence was highest in HIV−/PrEP+ MSM (40.3%), followed by HIV+ (30.8%) and HIV−/PrEP- (25.0%). The single prevalences for the different pathogens and anatomical sites showed a similar pattern (see Figs. 2 and 3). The prevalence for all tested pathogens and locations was highest in HIV−/PrEP+. While the prevalence for pharyngeal and urogenital infections was similar in non-PrEP users and HIV+ MSM (*p* < 0.05), the prevalence for rectal infections was higher in HIV+ MSM.

### Independent risk factors for STI

The final multivariable regression model on the effect of the three risk groups on being tested positive for at least one STI included age, city of testing and country of birth. Being HIV+ (OR 1.7, 95%-CI 1.3–2.2) or using PrEP (OR 2.0, 95%-CI 1.5–2.7) were independent risk factors, also partly younger age groups (Table 3).

**Table 3:** Independent risk factors for STI-acquisition regarding risk groups, bivariable and multivariable logistic regression (*n* = 2145)

	Bivariable analysis			Multivariable analysis*		
	OR	95%-CI	p	OR	95%-CI	*p*
Risk group (ref. HIV−/PrEP-)						
HIV+	1.33	1.08–1.64	0.01	1.7	1.34–2.16	0.00
HIV−/PrEP+	2.03	1.51–2.70	0.00	1.98	1.46–2.66	0.00
Demographics						
Age groups (ref. 40–49 yrs)						
18–24 yrs	0.82	0.51–1.31	0.40	1.08	0.64–1.79	0.78
25–29 yrs	1.46	1.08–1.96	0.01	1.48	1.08–2.04	0.02
30–39 yrs	1.26	1.00–1.59	0.05	1.16	0.90–1.48	0.24
50–59 yrs	0.69	0.51–0.91	0.01	0.67	0.50–0.91	0.01
> 59 yrs	0.45	0.25–0.81	0.01	0.5	0.28–0.91	0.02
City of testing (ref. Cologne)						
Aachen	0.48	0.36–0.91	0.02	0.68	0.42–1.09	0.11
Berlin	1.2	0.88–1.63	0.26	1.22	0.87–1.70	0.24
Bochum	0.58	0.39–0.85	0	0.66	0.44–1.0	0.05
Dortmund	0.77	0.46–1.28	0.31	0.91	0.54–1.54	0.74
Dresden	1.00	0.61–1.62	0.99	1.07	0.65–1.77	0.79
Munich	1.19	0.82–1.73	0.94	1.15	0.78–1.71	0.48
Nurnberg	1.56	0.73–3.32	0.25	1.53	0.69–3.40	0.29
Stuttgart	0.99	0.66–1.49	0.96	1.03	0.67–1.59	0.88
Country of birth (ref. Germany)						
Other country	1.24	1.01–1.51	0.04	1.06	0.85–1.32	0.60

* *p* < 0.01 for overall multivariable logistic regression model

The regression model on the effect of HIV in MSM not using PrEP (HIV+ MSM vs. HIV−/PrEP- MSM) on being tested positive for at least one STI included age, city of testing and country of birth and showed HIV+ as independent risk factor (OR 1.8, 95%-CI 1.4–2.3; Additional file 2: Table S1). The likewise model on the effect of PrEP use in HIV- MSM included the same variables and showed PrEP use as independent risk factor (OR 2.0, 95%-CI 1.5–2.7; Additional file 1: Table S2).

The regression model on the influence of sexual behavior on being tested positive for at least one STI included the variables age group, city of testing, number of male sexual partners, sex without condom, and the use of party drugs. Independent risk factors were having more than five male sex partners within the last 6 month (OR 1.6, 95%-CI 1.2–2.0), having sex without using a condom within the last 6 month (OR 2.1, 95%-CI 1.6–2.8) and the use of party drugs within the last 6 months (OR 1.6, 95%-CI 1.3–2.0; Table 4). Younger age was an independent risk factor, partly significant, partly nearly reaching level of significance (Table 4).

**Table 4:** Independent risk factors for STI-acquisition regarding sexual behaviour, bivariable and multivariable logistic regression (*n* = 1864)

	Bivariable analysis			Multivariable analysis*		
	OR	95%-CI	p	OR	95%-CI	p
Demographics						
Age in groups (ref. 40–49 yrs)						
18–24 yrs	0.82	0.51–1.31	0.40	1.10	0.64–1.85	0.33
25–29 yrs	1.46	1.08–1.96	0.01	1.45	1.02–2.07	0.04
30–39 yrs	1.26	1.00–1.59	0.05	1.28	0.97–1.67	0.08
50–59 yrs	0.69	0.51–0.91	0.01	0.72	0.51–1.01	0.06
> 59 yrs	0.45	0.25–0.81	0.01	0.60	0.30–1.17	0.13
City of testing (ref. Cologne)						
Aachen	0.77	0.36–0.91	0.02	0.89	0.53–1.50	0.66
Berlin	1.20	0.88–1.63	0.26	1.10	0.76–1.60	0.60
Bochum	0.58	0.39–0.85	0	0.72	0.45–1.13	0.16
Dortmund	0.77	0.46–1.28	0.31	1.50	0.82–2.74	0.19
Dresden	1	0.61–1.62	0.99	1.84	1.03–3.29	0.04
Munich	1.19	0.82–1.73	0.94	1.37	0.89–2.11	0.15
Nurnberg	1.56	0.73–3.32	0.25	1.71	0.68–4.30	0.25
Stuttgart	0.99	0.66–1.49	0.96	1.24	0.77–1.98	0.38
Sexual behaviour during the last 6 months						
Number of male sex partners (ref. 0–5)						
> 5	2.12	1.74–2.58	0.00	1.56	1.25–1.96	0.00
Sex without condom (ref. no)						
yes	2.70	2.13–3.42	0.00	2.09	1.58–2.76	0.00
Use of party drugs (ref. no)						
yes	2.68	2.02–3.57	0.00	1.62	1.30–2.01	0.00

* *p* < 0.01 for overall multivariable logistic regression model

## Discussion

The MSM Screening Study enabled us to picture the STI epidemiology in an extensive sample of MSM in Germany during a period of large-scale PrEP implementation. Overall, nearly one of three MSM was diagnosed with at least one of the tested STI, and the prevalence was significantly higher in PrEP users.

### STI prevalences

With 17.0%, the prevalence of MG nearly doubled that of CT or NG. Currently, there is only limited data available on MG in MSM. A meta-analysis found much lower prevalence estimates for MSM of 3.2% in five community-based studies from Australia and Central America and 3.7% in four clinic-based studies from Europe and the US [35]. Other studies found MG prevalences between 2.0 and 13.4%, and differed in the number of tested sites, clinical status, and reported sexual behavior [36–42].

In comparison to results of the hitherto existing studies, the MSM Screening Study conducted in 2018 found one of the highest MG prevalences in MSM reported, in particular for pharyngeal MG infections, which are reported to be rare in previous studies [34, 39, 40, 43], but also for anorectal infections. The high MG prevalence in our study is of special interest. We deliberately recruited at MSM friendly practices with a general practitioner profile and not only serving as sexual health centers. Therefore, we could recruit a nationwide large sample of a more general MSM population and not only MSM with distinct high sexual risk behavior. As a result, we expected the STI prevalences in our study to be lower than those found in studies conducted in specialized STI testing facilities. Possible reasons for the lower prevalences in previous studies could be that testing was only performed in one or two localizations, study populations had lower risk profiles, or general epidemiological differences by person, place and time. Whether the increasing MG prevalence in more recent studies is a real trend or due to demographic, behavioural or clinical differences between the study populations remains unclear. Moreover, test sensitivity may play a role, as RNA targeting Aptima TMA technology usually shows higher sensitivity for STI than DNA targeting PCR based assays used in some previous studies.

The overall prevalence of CT (10.1%) and NG (8.6%) in our study were lower than for MG, but still high, and comparable to other studies among MSM, especially in Western countries. Globally, prevalences varied between 1 and 24% for CT, and 0 and 54% for NG [8, 11, 44–55], depending on the type of the recruiting institution, clinical symptoms, HIV status and sexual behaviour of the participants. Extragenital as well as asymptomatic infections were reported to be common.

For NG, a distinct higher proportion of the overall prevalence was diagnosed pharyngeal. Despite a higher rate of spontaneous clearance and a shorter persistence of NG in the throat than in other localizations, this higher proportion of pharyngeal NG is of special concern, as the pharynx is an important reservoir for the development of antimicrobial resistance (AMR) of NG [56]. To eliminate the often asymptomatic pharyngeal NG as a transmission reservoir as well as to reduce the development of AMR, antiseptic mouthwash as a non-antibiotic preventive intervention has been suggested [57–59], but efficacy has yet to be established.

The high proportion of extragenital and asymptomatic infections in our study draws the attention to their high impact for an ongoing transmission of STI in MSM population by not diagnosing and treating them effectively [12, 39, 40]. The WHO recommends to have respective screening offers for MSM if the prevalence of asymptomatic pharyngeal and rectal infections exceeds 1 to 2% [60].

By only testing the participants for urogenital STI in our study, we would have only found 27.7% of all diagnosed infections. If no general screening offers for MSM would be available, only symptomatic MSM would attend the practices for STI testing. To assess the impact of clinical symptoms for an effective STI care in MSM, we used information from self-reported STI-related symptoms. By exclusively testing MSM reporting STI-related symptoms, only 31.0% of all diagnosed STI would have been identified. The proportion of missed MG diagnoses would have been the highest in this context.

Given the high overall prevalences of CT, MG and NG in our study and the high proportions of extragenital and asymptomatic infections, the results strongly support broadly implemented STI-screening offers for MSM with special emphasis of screening at all three localizations.

We found only two infections with TV in the study population, which corresponds with low prevalence also found in other comparable studies on TV in MSM. A low prevalence in MSM may be due to a general higher persistence of this pathogen in the female urogenital tract [61–63]. On basis of the study results, the inclusion of TV in a regular STI testing scheme for MSM is not recommend.

Although syphilis is an important STI among MSM [2, 13, 64], the need for drawing additional blood might have led to a decrease in participation and have reduced the power of the results. Therefore, we did not test for syphilis in our study.

### Risk factors for STI

A substantial number of PrEP users participated in the MSM Screening Study. The prevalence for each of the tested STI and at each localisation was highest among PrEP using HIV- MSM. PrEP users also reported distinct higher sexual risk behaviour. Additionally, PrEP use was an independent risk factor for diagnosis of STI in the multivariable model.

In contrast to previous studies, we found no difference in STI prevalence between HIV+ and HIV- MSM [13, 14] on a descriptive level. Compared to other studies, this was more due to a comparably higher STI prevalence in HIV- MSM than to a lowered prevalence in HIV+, resulting from a very high prevalence in PrEP using HIV- MSM. Accordingly, HIV+ MSM had a higher risk for STI compared to HIV- non-PrEP users in the respective multivariable model. The very low risk of HIV transmission while having a successfully supressed HIV infection could lead to a higher sexual risk behaviour and therewith a higher prevalence of STI. Anyway, we did not find such difference comparing the overall STI prevalence between HIV+ MSM having HIV viral load below or above the detection limit but this may be due to the small proportion of HIV+ MSM with viral load above the detection limit (15.8%).

Besides PrEP use and HIV-status, we also identified other relevant risk factors for being tested positive for an STI. Those were behavioral factors and included reporting condomless sex, having had more than five male sex partners and using party drugs, all within the last 6 months. These risk factors were also found in several other studies [3, 6, 7, 14]. Our study complements the results of a recent meta-analysis and other current cross-sectional studies that showed an association between PrEP use and STI diagnosis [30], and reported PrEP use as important risk factor for STI diagnosis [27, 40, 65]. A large longitudinal study from Australia could even show increasing STI incidences after initiation of PrEP [29]. A higher STI testing frequency after commencing PrEP might be a confounder for higher STI prevalences in PrEP users, but the respective study adjusted for STI testing frequency and recent a study from the US showed that an increase of STI prevalence in PrEP using MSM was independent from a concurrent increase of STI testing in this group [66]. Despite the concerns about rising STI incidence due to PrEP, the clear association between PrEP use and STI diagnosis in our study also shows that PrEP reaches the right persons having a demand for this HIV-preventional measure.

Against this background, regular STI testing of PrEP users is an important measure to detect STI, to minimise the risk of sequelae on the individual level, and to eradicate relevant transmission reservoirs on the Public Health level. With the recently introduced cost coverage of PrEP and accompanying STI tests by the compulsory health insurance in Germany, the number of PrEP users might increase probably and therewith the number of STI diagnoses. The prediction by Jenness et al. [32], that the incidence of STI in PrEP users will decrease due to effective screening and treatment measures, cannot be answered for Germany currently. Monitoring the STI epidemiology in the context of PrEP use further will therefore be of special importance.

As our study design was cross sectional, we were not able to analyse if MSM using PrEP showed higher sexual risk behaviour due to their PrEP use, or if they decided to use PrEP due to their sexual risk behaviour as a risk minimisation strategy. However, considering the sexual risk profile of PrEP users in our study, the results showed clearly that PrEP reached the right persons showing a demand for PrEP due to their sexual risk profile. Besides this, we found high STI prevalences and relevant sexual risk behaviour also in HIV- non-PrEP users and HIV+ MSM. This highlights the need for appropriate risk-adapted STI testing and treatment programs for all MSM. In this context, an effective medical history regarding sexual health, risk and health seeking behaviour is an important basis for delivering high quality and evidence based STI services to the relevant populations. To reach as many persons as possible, low-threshold and low or free of cost preventive, diagnostic, and treatment offers for STI for MSM should be broadly available. Innovative testing offers including possibilities for online communication and self-sampling should complement existing local structures.

### Clinical considerations

The screening frequency for CT, NG and syphilis in asymptomatic MSM is discussed in various guidelines for different groups: HIV+ MSM are recommended to be screened annually, PrEP users and MSM with changing partners every 3–6 months. Our study suggests that having more than 5 male sex partners in the last 6 months, having sex without using a condom, using party drugs, and being HIV+ or using PrEP are the most important risk factors for MSM to acquire an STI. Therefore all MSM reporting one or more of these items should be screened every 3–6 months. In clinical practice it is a challenge to modify screening strategies according to risk factors. Often simple algorithms (e.g. to screen every HIV+ patient once a year) are used. A structured questionnaire or score on basis of the risk factors found could be used to allocate resources more effectively.

With the introduction of PrEP and the challenge of additional STI care, the questions of potential over- as well as under-treatment gains high impact. It is widely accepted that all symptomatic cases of STI should be treated (including MG). Resistance testing to avoid AMR in NG is important and a culture swab should be taken before any GO treatment, but in clinical practice in only less than 40% cultures yield successful results.

Asymptomatic STI are common in pharyngeal and rectal infections. Oro-penile and oro-anal sex as well as the use of saliva are relevant for the transmission of STI, particularly for gonorrhoeae [66, 67]. Spontaneous clearance of CT and NG has been reported [67] but sexual abstinence for a not defined time is not an option for most clients. Even though they can be self-limiting, ECDC and WHO recommend treatment of all asymptomatic pharyngeal NG infections due to their high potential of generating AMR in this localisation through genetic exchange with commensal pathogens. Against this background, all detected infections with NG, but also CT should be treated and a test of cure should be performed to avoid hidden transmission reservoirs.

MG screening and treatment of asymptomatic MSM is highly debated not only due to reported genotypic resistance to the standard treatment with azithromycin of up to 80%, but also because of the partly high prevalence of MG and the possible damage of repeated antibiotic treatment on the microbiome (Read et al., 2019). The collateral damage to resistomes [68–70] is already done and cannot be reduced by ignoring wide spread of resistant MG infections.

In Germany testing for MG macrolide resistance is not yet a standard that is reimbursed by compulsory health insurance and the best treatment of azithromycin resistant strains is also not clear. Therapy guidelines recommend the alternative use of Moxifloxacin [71], but using gyrase inhibitors is limited by side effects, and resistance is increasingly being reported in Germany as well [72]. However, resistance testing for quinolones is not widely available yet. On the other hand many cases with macrolide resistant mutations can still be treated with higher azithromycin doses or consecutive therapy of doxycycline and azithromycin [71]. Assumption of costs for pristinamycin is not assured in Germany, because it is only available from international pharmacies.

MG resistance against azithromycin is more common in MSM than in heterosexual men [73]. This is probably due to the more frequent exposure of asymptomatic mycoplasma infections to azithromycin when treating CT or GO: 12% of participants tested STI positive in our study had a concurrent infection of MG with CT, NG, or both. Consequently, a test for MG should be considered before treatment of CT or GO to identify coinfections and avoid ineffective MG co-treatment and undetected MG resistance. In this context, current technical developments in terms of MG resistance testing at the clinical site could be of importance.

Generally, the STI panel of currently available commercial multiplex test kits is not based on clinical usefulness and include too many facultative pathogens (e.g. ureaplasma) or pathogens with no clinical implication (e.g. mycoplasma hominis, cytomegalovirus). In case of using these kits a good communication of the relevance of positive test results for the specific pathogens is important. Not reporting clinically irrelevant positive results is not only a legal issue, it is also a confession of failure of education of medical personnel and clients. New multiplex tests should be developed, that cover only pathogens in clinically relevant combinations.

Partner notification is an important tool to interrupt infection chains. In groups with many changing partners and good communication this can lead to a high frequency of prophylactic antibiotic use if notified partners are treated immediately before getting their specific test result, as recommended for infection with CT and NG. Further studies have to show, if this practice of partner treatment before testing should also be applied to PrEP users. The context of counselling and preparing eligible persons for PrEP is an excellent chance to screen for STI and to sensitize for the transmission risks and consequences of antibiotic treatment.

### Limitations

Our study has several limitations. We recruited a convenience sample of MSM, so the results cannot be generalized to all MSM living in Germany. The large proportion of higher educated among HIV-negative participants compared to HIV-positive indicates that we experienced a selection bias. By recruiting participants through infectiologically specialised practices, we might have reached a more general sample of HIV-positive MSM. They are attending these type of practice more frequently due to their underlying chronic disease, while HIV-negative MSM might visit these practices more often if being better informed about this specialised services, despite they often act as general practitioners for MSM.

Nevertheless, as we recruited nationwide a large sample of MSM through a comprehensive network of MSM friendly practices with infectiological focus and also serving as general practitioners for MSM, we consider that we could draw an epidemiological picture of a relevant part of the MSM community in Germany.

By recruiting MSM via medical practices, a recruitment bias towards MSM with higher probability of having a STI could be probable. As only 32% of study participants tested positive for any STI reported also STI-related symptoms in the previous 4 weeks, the study approach to reach a more general MSM population seemed to be successful. By asking detailed questions on sexual behavior, a reporting bias could occur. We do not consider this as very probable, as intimate questions such as on sexual risk behaviour and drug use were answered thoroughly by the participants and specific answers were not avoided. At last, a cluster effect could influence the analyses occurring due to specific patient populations of single study sites e.g. patients with comparably high sexual risk profile. This could possibly lead to over- or underestimation of the STI prevalence, but the multivariable model was adjusted for that.

## Conclusions

In our study, we found a high STI prevalence in MSM in Germany; the prevalence of MG was especially high. STI were mainly asymptomatic, and with urogenital screening we would have only found 27.7% of all diagnosed STI. HIV/PrEP-status, having more than 5 sex partners, having condomless anal intercourse (insertive and/or receptive) and the use of party drugs were independent risk factors for STI diagnosis.

Risk adapted, comprehensive, multi-localisation and highly frequent STI testing for MSM using PrEP and beyond should be available, assuring testing options with low threshold and free of cost. This seems to be essential to facilitate early treatment and reduce further spread. Counselling of PrEP users should address regular STI testing and the risk of using party drugs. Antibiotic stewardship is important to avoid antibiotic resistance in frequently infected and co-infected patients.

## Supplementary information

**Additional file 1.** Questionnaire MSM Screening Study

**Additional file 2:** **Table S1.** Independent risk factors for STI-acquisition regarding HIV-status, multivariable logistic regression model (*n* = 1, 873)

**Additional file 3:** **Table S2.** Independent risk factors for STI-acquisition regarding PrEP use, multivariable logistic regression model (*n* = 1, 006)

## Data Availability

The dataset supporting the conclusions of this article is available in the Zenodo repository (https://zenodo.org), accession number 3407406.

## Abbreviations

AMR: Antimicrobial resistance
CAI: Condomless anal intercourse
COI: Condomless oral intercourse
CT: Chlamydia trachomatis
HIV: Human immunodeficiency virus
MG: Mycoplasma genitalium
MSM: Men who have sex with men
NG: *Neisseria gonorrhoeae*
PrEP: HIV Pre-exposure prophylaxis
STI: Sexually transmitted infections
