# Supplementary material for: STI in times of PrEP: high prevalence of chlamydia, gonorrhea, and mycoplasma at different anatomic sites in men who have sex with men in Germany
Source: BMC Infect Dis. 2020 Feb 7;20:110. doi: 10.1186/s12879-020-4831-4 (PMC7007644; doi:10.1186/s12879-020-4831-4)
Supplement: Supplementary file 1 — Additional file 1. Questionnaire MSM Screening Study [file 12879_2020_4831_MOESM1_ESM.pdf]

ROBERT KOCH INSTITUT

labor krone

Questionnaire MSM Screening Study

1. **What year were you born?**
☐☐☐☐

2. **Where do you live?**
(first three numbers of your postal code)
☐☐☐

3. **Were you born in Germany?**
☐Yes ☐No. Country of origin: _____________

4. **What is your highest school degree?**
☐I am still in school
☐I haven´t graduated high school
☐9th grade
☐10th grade
☐High school diploma

5. **What kind of relationship do you currently have?**
☐I have a monogamous relationship
☐I have an open relationship with an agreement for sex with others
☐I have an open relationship without an agreement for sex with others
☐I am single/I don´t have a relationship
☐Other kind of relationship
☐I don´t know

6. **How many sex partners did you have in the last 6 months?**
___ men
___ women
☐I don´t know
☐I didn´t have sex

7. **What kind of relationship did you have with your sex partners in the last 6 months?**
(More than one answer possible, if you had multiple sex partners)
☐Only steady sex partners
☐Only non-steady sex partners
☐Both, steady and non-steady sex partners
☐I don´t know

8. **Where did you meet your sex partners in the last 6 months?**
(More than one answer possible, if you had multiple sex partners)
☐Internet
☐Pub, bar, nightclub
☐Sauna
☐Sex party
☐Porn cinema
☐Park/ cruising location
☐Through friends
☐Classified advertising
☐Brothel
☐Street
☐Street prostitution
☐Other places
☐I don´t know

9. **Did you have anal/vaginal sex without a condom in the last 6 months**
☐Yes ☐No ☐I don´t know

9.1. **If yes, with how many sex partners did you have sex without a condom in the last 6 months?**
___ men
___ women
☐I don´t know

10. **In the last 6 months, what kind of sex did you have with your sex partners?**
(more than one answer possible)
☐Anal sex with condom
   ☐active ☐passive
☐Anal sex without condom
   ☐active ☐passive
☐Oral sex with condom
   ☐active ☐passive
☐Oral sex without condom
   ☐active ☐passive
☐Rimming (ass licking)
   ☐active ☐passive
☐Fisting (put the fist in the others ass)
   ☐active ☐passive
☐others __________
☐I don´t know

11. **Did any of your sex partners use saliva as lubricant before inserting his finger, penis or other objects into your bottom?**
☐Yes ☐No ☐I don´t know

12. **In the last 6 months, did you use alcohol or drugs before or while having sex?**
☐Yes ☐No ☐I don´t know

12.1. If yes, what kind of?
(More than one answer possible)
☐Alcohol
☐Cannabis
☐Viagra/Cialis
☐Poppers
☐Cocaine
☐Ecstasy
☐Speed
☐Crystal Meth
☐GHB/GBL
☐Bath salts/ Spice
☐Others
☐I don´t know

13. If you have anal/vaginal sex without a condom: Do you use other strategies to reduce the risk of a HIV infection?
(More than one answer possible)
☐I don´t have sex without a condom.
☐I only have sex without a condom in monogamous relationships.
☐I only have sex without a condom if I am the active partner.
☐ I only have sex without a condom if I am the passive partner.
☐I tell my sex partner not to ejaculate inside my bottom.
☐I try to assess if my sex partner is HIV positive.
☐I ask my sex partner for his HIV status
☐If my sex partner is HIV positive, I only have sex with him/her if he/she has an undetectable viral load.
☐I only have sex without a condom with healthy looking men.
☐I use PrEP (pre-exposure prophylaxys)
☐ My sex partner uses PrEP (pre-exposure prophylaxys)
☐I think my HIV risk is low because I´m circumcised.
☐I use other strategies.
☐No, I don´t use other strategies.
☐I don´t know

14. In the last 6 months, did you have non-steady sex partners…
14.1. who you paid to have sex with you?
☐Yes ☐No

14.2. who paid you to have sex with them?
☐Yes ☐No

15. Have you ever been tested for one of the following infections?
(More than one answer possible)?
☐HIV
☐Syphilis
☐Hepatitis B
☐Hepatitis C
☐Chlamydia/ LGV
☐Gonorrhoea
☐Mycoplasma
☐Trichomonas
☐I don´t know
☐No

16. Have you ever been diagnosed with one of the following infections?
(More than one answer possible)
☐HIV
☐Syphilis
☐Hepatitis B
☐Hepatitis C
☐Chlamydia/LGV
☐Gonorrhoea
☐Mycoplasma
☐Trichomonas
☐I don´t know
☐No

17. Have you ever been treated for one of the following infections?
(More than one answer possible)
☐HIV
☐Syphilis
☐Hepatitis B
☐Hepatitis C
☐Chlamydia/LGV
☐Gonorrhoea
☐ Mycoplasma
☐ Trichomonas
☐I don´t know
☐No

18. In the last 4 weeks, did you have one or more than one of the following physical symptoms?
-pain when urinating
-Itching/pain of the penis, bottom or throat
-skin lesions at penis, bottom or throat
-penile or anal discharge
☐Yes
☐No
☐I don´t know

19. If you are HIV positive:

19.1. When were you first diagnosed HIV positive

☐Within the last 6 months
☐Within the last 12 months
☐More than 12 months ago
☐I don´t remember

19.2. What was your last viral load?

☐below threshold
☐<1,000 copies/ml
☐1,000 – 10,000 copies/ml
☐10,000 – 100,000 copies/ml
☐>100,000 copies/ml
☐I don't know

19.3. Do you take any HIV medication (HIV combination therapy)?

☐Yes ☐No ☐I don´t know

20. If you are HIV negative: Do you currently take PrEP (pre-exposure prophylaxis) against HIV?

☐Yes ☐No ☐I don´t know

Thank you for taking part!
