# Supplementary material for: STI in times of PrEP: high prevalence of chlamydia, gonorrhea, and mycoplasma at different anatomic sites in men who have sex with men in Germany
Source: BMC Infect Dis. 2020 Feb 7;20:110. doi: 10.1186/s12879-020-4831-4 (PMC7007644; doi:10.1186/s12879-020-4831-4)
Supplement: Supplementary file 2 — Additional file 2: Table S1. Independent risk factors for STI-acquisition regarding HIV-status, multivariable logistic regression model (n = 1, 873) [file 12879_2020_4831_MOESM2_ESM.docx]

|  | **Multivariable analysis*^,^**** | | |  |
| --- | --- | --- | --- | --- |
|  | **OR** | **95%-CI** | **p** |  |
|  |  |  |  |  |
| **HIV status (ref. HIV-)**  HIV+ | 1.80 | 1.42-2.30 | 0.00 |  |
|  |  |  |  |  |
| **Demographics** |  |  |  |  |
| **Age in groups (ref. 40-49 yrs)** | |  |  |  |
| 18-24 yrs | 1.26 | 0.73-2.15 | 0.41 |  |
| 25-29 yrs | 1.83 | 1.29-2.58 | 0.00 |  |
| 30-39 yrs | 1.12 | 0.85-1.467 | 0.42 |  |
| 50-59 yrs | 0.73 | 0.54-1.00 | 0.06 |  |
| >59 yrs | 0.48 | 0.25-0.90 | 0.02 |  |
|  |  |  |  |  |
| **City of testing (ref. Cologne)** | |  |  |  |
| Aachen | 0.76 | 0.46-1.23 | 0.29 |  |
| Berlin | 1.50 | 1.04-2.17 | 0.03 |  |
| Bochum | 0.72 | 0.56-1.13 | 0.15 |  |
| Dortmund | 1.04 | 0.61-1.75 | 0.14 |  |
| Dresden | 1.21 | 0.72-2.02 | 0.48 |  |
| Munich | 1.40 | 0.90-2.18 | 0.14 |  |
| Nurnberg | 1.90 | 0.84-4.32 | 0.13 |  |
| Stuttgart | 1.19 | 0.75-1.87 | 0.46 |  |
|  |  |  |  |  |
| **Country of birth (ref. Germany)** | |  |  |  |
| Other country | 1.07 | 0.83-1.36 | 0.62 |  |

*PrEP users excluded from this analysis

** p<0.01 for overall multivariable logistic regression model
