# Supplementary material for: STI in times of PrEP: high prevalence of chlamydia, gonorrhea, and mycoplasma at different anatomic sites in men who have sex with men in Germany
Source: BMC Infect Dis. 2020 Feb 7;20:110. doi: 10.1186/s12879-020-4831-4 (PMC7007644; doi:10.1186/s12879-020-4831-4)
Supplement: Supplementary file 3 — Additional file 3: Table S2. Independent risk factors for STI-acquisition regarding PrEP use, multivariable logistic regression model (n = 1, 006) [file 12879_2020_4831_MOESM3_ESM.docx]

| **Multivariable analysis*^,^**** | | | | | | |
| --- | --- | --- | --- | --- | --- | --- |
|  | | **OR** | | **95%-CI** | | **p** |
|  | |  | |  | |  |
| **PrEP-use (ref. no)**  yes | | 1.97 | | 1.45-2.67 | | 0.00 |
|  | |  | |  | |  |
| **Demographics** | |  | |  | |  |
| **Age in groups (ref. 40-49 yrs)** |  | |  | |  |  |
| 18-24 yrs | | 0.88 | | 0.47-1.63 | | 0.68 |
| 25-29 yrs | | 1.24 | | 0.79-1.94 | | 0.35 |
| 30-39 yrs | | 1.09 | | 0.75-1.58 | | 0.67 |
| 50-59 yrs | | 0.59 | | 0.32-1.07 | | 0.08 |
| >59 yrs | | 0.76 | | 0.19-3.00 | | 0.70 |
|  | |  | |  | |  |
| **City of testing (ref. Cologne)** |  | |  | |  |  |
| Aachen | | 0.73 | | 0.30-1.78 | | 0.49 |
| Berlin | | 0.85 | | 0.49-1.48 | | 0.57 |
| Bochum | | 0.50 | | 0.26-0.98 | | 0.04 |
| Dortmund | | 0.81 | | 0.20-3.36 | | 0.77 |
| Dresden | | 1.53 | | 0.34-6.90 | | 0.58 |
| Munich | | 0.79 | | 0.42-1.47 | | 0.45 |
| Nurnberg | | 1.34 | | 0.35-5.19 | | 0.67 |
| Stuttgart | | 0.59 | | 0.23-1.47 | | 0.26 |
|  | |  | |  | |  |
| **Country of birth (ref. Germany)** |  | |  | |  |  |
| Other country | | 1.22 | | 0.09-1.65 | | 0.20 |

*HIV+ participants users excluded from this analysis

** p<0.01 for overall multivariable logistic regression mode
